# Account of an Extraordinary Cure of a Wound of the Intestines

**Published:** 1807-12

**Authors:** John Redman Coxe


### *Account of an extraordinary Cure of a* Wound *of the* Intestines.

[ Extracted from the Philadelphia Medical Museum. ]

THE following account of an extraordinary recovery from a wound of the intestines, was sent me some years ago by a relation in London, to whom it was given by a friend; but no information was afforded as to the author, or by what means the paper came into his hands.—As, however, the author has adverted to his connection with the Radcliffe Infirmary, Oxford, I have concluded it might be useful to publish the case, as its truth may be perhaps established by this measure.

JOHN REDMAN COXE, M. D.

---

In the evening of the 26th of September, 1775, I was called in great haste, to James Langford, a young man in the 21st year of his age, who had been maliciously stabbed with a knife in the left side of his belly. The wound was between two and three inches in length, running from the left *os ilium* obliquely upwards towards the navel. I found him lying on the floor, weltering in his blood, with a large portion of his intestines forced through the wound; and I learnt, from the unfortunate youth himself, that, as soon as the wound was inflicted, the bowels began to appear; and, by the time I got to him, which could not exceed ten minutes, I verily believe, that the full half of the intestinal tube was protruded through the opening. This I attributed, in some measure, to the fulness of the stomach; for, immediately before the accident happened, he had eaten a very hearty supper. The wound at first bled freely; but the *hemorrhage* was soon restrained by the pressure of the prolapsed intestines, which were, to a great degree, distended with air; and from this circumstance I was flattered with the best hopes that they had escaped the assassin's knife; but, to my great disappointment, it proved otherwise, as will appear most evidently from the sequel of this narrative. Examining his pulse, I found it was exceedingly low, quick, and interrupted; his skin was all over cold and clammy, and he laboured under great languor, anxiety, and pain about the *præcordia*. He likewise complained of a disagreeable tingling and numbness of the whole thigh, leg, and foot, of the side wounded; and acquainted me, that he dropped on the floor, in consequence of the inability of the limb to support him, and not from any faintness, as might have been reasonably expected, from the loss of blood, or througn fear, to which, indeed, he seemed an utter stranger. I ordered him to be conveyed to his bed in an horizontal posture, lest the raising of the body might encourage a farther descent of the parts, which still remained in the *abdomen*; and a fomentation of port wine, with warm water, to be got ready immediately, out of which a double flannel should be wrung, and applied directly to the prolapsed intestines, and renewed occasionally, to prevent them from getting too dry, as well as to preserve, as much as possible, their natural heat. The reduction of the displaced bowels was begun, with laying the patient's legs over an assistant's shoulders, who was desired to kneel upon the bed for that purpose, with his back towards him, and then the legs were brought forward as far as to the hams. By these means, the lower parts of the body were elevated, and, in consequence, the weight of the bowels falling back towards the chest, counteracted their further protrusion. While the patient continued in this position, I endeavoured, with my hands, to force the bowels back into their proper place; but soon found, from the quantity of them protruded, together with their great inflation, that a larger, or more extended pressure than my own hands could afford me, was necessary; and not thinking it prudent to employ any of the bystanders in so hazardous a task, lest, by their inexperience they might handle the bowels too roughly, I sent for two of my fellow-labourers in the care of the Radcliffe Infirmary, to my assistance. As soon as they came, the reduction was again attempted; one of us directing that portion of the bowel which was last protruded, while the two others made a gentle, regular, and circumscribed pressure from all sides towards the opening. But this endeavour not succeeding, convinced us, that it would be much safer to enlarge the wound, to facilitate the return of the prolapsed parts, than hazard the necessity of handling them too much, or exposing them too long to the circumambient air, either of which would, in all probability, have proved fatal. This being done accordingly, by continuing the wound in the same direction upwards, about two inches, the exposed bowels were easily and soon returned into the *abdomen*. We then brought the edges of the wound together, and kept them by the suture called *gastroraphia*, leaving a proper opening in the most depending part of it, for the discharge of the blood or matter which might be collected in the cavity; and afterwards it it was dressed in the usual way, lightly, and almost superficially, with an anodyne poultice over all.

The regimen enjoined him, with respect to diet, was only gruel, panada or sago; with barley water or thin gruel to drink; and the medicines were the following:

℞. Mannæ. Ol. Amygd. D. anna ℥ß. Aq. Alexit. simp. ℥i.—Nucis moschat. ʒi.——M. ft. haustus quamprimum sumendus et quartâ quâque horâ repetendus donec alvus responderit.

℞. Decoct. Pectoral. ℥viij. Ol. Lini ℥ij. Tinct. Thebaic. gt. xl.——ft. enema quovis tempore infundendum si dolor abdominis urgeat.

27th. Visiting him early the next morning, I found the night had been spent in great restlessness and inquietude, notwithstanding the clyster had been thrown up according to the direction. He was exceeding low; his skin felt still cold and clammy; his pulse weak and fluttering; he complained of frequent chills, and an oppressive tightness of his belly, though the wound had discharged considerably a thin, serous humour, which had wetted the bandage quite through. Nor was the tension of the *abdomen*, at this time, sufficient to account for the oppressive pain he complained of; from whence I concluded it to be spasmodic.

The dressings were removed; and I desired my apprentice to foment the part with an infusion of the emollient flowers, for a full hour, and to take particular care that the stupes were applied of a very moderate warmth; often having observed, that this manner of applying them, when an inflammation was either to be resolved or prevented, was more effectual than when the heat has been greater. This observation, upon a little reflection, will be found agreeable to reason; for as great heat proves an astringent, on the contrary, a moderate and kindly warmth relaxes, and, by promoting a free perspiration of the parts to which it is applied, sooner effects the end proposed. The wound was dressed as before, with the addition of two ounces of the species procataplasmate de cymino to the poultice; and, as the draughts he had taken had not produced any motion of the bowels, it was thought proper to inject the underwritten clyster, as soon as it could be prepared:

℞. Decoct. pro Clyst. ℥viij. Ol. Lini. ℥ij. Elect. Lenitiv. Mel. Solutiv. ana ℥ß.——ft. enema.

This, in about half an hour, occasioned a very copiou discharge of *fæces*, together with a good deal of *blood*; some of it congealed into lumps, the rest fluid. This cir-

 cumstance cumstance did not fail to alarm my apprehensions of the imminent danger of the lad's situation, as it was no longer to be doubted, but that the bowels were wounded in some part of them; but what part still remained a matter of conjecture. When the clyster had done operating, he took this draught:

℞. Sperm. Ceti, (Mucilag. Gum. Arab. Solut.) ℈i. Aq. Alexit. Simp. ℥iß. Ol. Amygd. D. Syr. de Meconio ana. ʒij. Tinct. Thebaic. guttas x.——M. ft. haust.

Late this evening the fomentation and dressings were again renewed, and directions given, that he should take one of the draughts, with manna, oil, &c. as first prescribed, at three o'clock in the morning; and to repeat them regularly every fourth hour, till they had had their desired effect.

28th. He had got but little sleep in the night, though he had lain something quieter, with short, but interrupted slumbers intervening. His pulse, and all the other symptoms, were much in the same state as yesterday, excepting a general soreness of the *abdomen*, of which, at this time, he made great complaint, and more particularly about the wounded part. The whole belly was full and tense; and, when I struck it with my finger, it returned an *emphysematose* sound. The discharge from the wound was increased; it had stained the bandage of a deep reddish-brown colour, and was of a disagreeable smell. The draughts he had taken had not yet moved him; therefore, I desired they might be continued, according to the general direction; and that, in case any stools should come off, to put them by, separately, for my inspection. By the time I made my evening visit, he had had two motions; in the first there was a good deal of fluid blood; with the last, but little, no more than to give it a tinge. He was evidently relieved by the evacuation; was calmer and more composed; his pulse was rather more up, and his skin warmer. He said, he found himself lightsomer; and he was not so tight, and thought he breathed with more freedom. When I came to loosen the bandage, I was greatly surprised to find it daubed all over with the discharge; but, as soon as the dressing was removed, there was no evidence wanting to assure me, that this discharge was in part fæcal, not only from the colour and smell of it, but likewise from the sharp pain it had occasioned in passing through the wound. My hopes of his recovery now began to fail me; however I resolved to persevere, and act as though I was sure of success. After dressing, he was ordered to take take the anodyne draught, and to begin again the manna draughts, with oil, early in the morning.

29th. Before I came to visit him, he had had another motion; and the nurse informed me, that his night had been better than any of the preceding ones, he having slept, at different times, full three hours. His pulse was stronger, but remitting, and his skin inclining to perspire. The tongue was foul, and the water clear and pretty high coloured. In the stool, which had come off this morning, I did not find any blood, or in any he had afterwards during the time of his confinement. The wound had discharged a great deal, and was more inflamed; and the edges of it looked thick and ill-natured, and were ready to separate from each other. The tension of the belly still kept up, though I did not perceive that it had at all increased. The opening draughts were continued, one in six hours only, through the course of this day, which kept him sufficiently open; and the anodyne was repeated at ten o'clock this night.

30th. This morning, things wore but a melancholy aspect. His night had been restless, and his head confused, and he talked sometimes incoherently; his pulse was increased, though exceedingly irregular, and the skin felt hot and dry; he was thirsty, and complained of a great tightness, particularly about the region of the stomach; his countenance was hollow, the eyes being sunk, with a deadness in them not easily to be expressed. The wound had discharged very much, and it was extremely offensive. The edges of it were inverted, much swollen, and separated from each other considerably more than the preceding day. He likewise complained of a sharp, burning pain, deep in the wound, but could not express precisely where. As soon as the wound was dressed, the anodyne clyster was administered; and I desired, that he might have a small bason of the infusion of mint, with a knob of fine sugar, got ready for him as soon as possible, and that he would sip it down as warm as he could. At two o'clock this afternoon, he was seized suddenly with a most violent vomiting, and brought up a large quantity of bile. This I the more wondered at, as he had never made the least complaint of sickness, or *nausea*, from the time of his accident; for every thing he had taken, had sat easy upon his stomach. What he had brought up was of so dark a colour, that I imagined it was mixed with blood; but, upon a careful examination of it, found I was mistaken. When the vomiting was over, the nurse gave him a little more or the mint infusion; and, soon after, he fell into a sound sleep, which continued more than an hour. In the evening he was hot and uneasy, complaining of thirst, and a pain in his head; his pulse was increased, and his skin felt dry. The wound had made a prodigious discharge, which I observed always to increase, in proportion as the bowels were more or less loosened by the medicines he was taking; and, from the violent efforts of the *abdominal* muscles in the time of his vomiting, most of the stitches in the wound were broken, so that you might plainly see into the cavity of the *abdomen*. After dressing the wound, twelve ounces of blood were taken from the arm, and the anodyne draught was given to him soon after.

October 1st. I learnt, from the people about him, that for a few hours, after he had taken the *opiate*, he lay composed; but soon after midnight, he awaked in great hurry and confusion, complaining of his stomach and bowels, accompanied with convulsive twitchings of the tendons; and that, about five o'clock this morning, he brought up another large quantity of bile, which gave him great relief; for afterwards he lay perfectly easy, and got between two and three hours sleep. At nine o'clock, when I made my morning visit, I found him much refreshed, and without any kind of complaint. His pulse was full, but much steadier than it had been any time before, and his skin was open. The water he had made was turbid, though still high-coloured. The wound, indeed, made but an indifferent appearance; the edges of it were very sloughy, particularly the tendons of the oblique muscles, and so far receded from each other, as to make it necessary to divide the remaining stitches. The lower part of the wound, or that next to the *illium*, was beginning to digest, and the inflammation and tension of the belly to abate. The opening draughts, made a little warmer, were continued, which kept the bowels constantly and gently open. In the evening his pulse was rather increased; and I found that, some time in the afternoon, he had brought up a little more bile, though without any previous complaining. After dressing, I directed more blood to be drawn, and the *opiate* to be repeated.

2nd. The nurse acquainted me this morning, that her patient had had a very quiet night, and had slept many hours without intermission; that he had taken a sufficient quantity of nourishment, and that it had sat well upon his stomach. I found him cheerful, without any complaint, except that of hunger. His pulse was steady, his skin soft and and open, his tongue getting cleaner, and his water beginning to break. The discharge this morning from the sore, was exceedingly offensive, and when I had taken off the dressing, I was really astonished at the horrid appearance! The wound was burst open, in such a manner as to assume a circular form, and was rather more than three inches in the least diameter of it. In the base of this dreadful opening, there was nothing to be seen but the circumvolutions of the small guts; and how this amazing breach was to be restored, I could not easily conceive. Had any one taken a view of the wound at this time, who was unacquainted with the real progress of it, he must naturally have concluded, there had been a great loss of substance. The patient was dressed with thin pledgets of very fine unformed lint, moistened with the oil of the flowers of the *hypericum,* luke-warm, laid first upon the exposed bowels; afterwards the cavity was filled up lightly with the same sort of lint, dry; the edges were covered with a moderately warm digestive, and the whole secured with the uniting bandage; which bandage had been used from the very beginning, to prevent, as much as art could prevent, the impending mischief.

3d. Appearances this morning were very favorable; he had slept well most part of the night; his pulse was perfectly quiet, and his skin moderately open. The water was become better coloured, and had made a fair separation; so that, from this time, all signs of fever, inflammation, and pain, its concomitant, entirely ceased: nor did there ever arise any alarming, or even disagreeable symptom, afterwards; but every thing went on in an easy, regular way. The wound digested kindly, and was constantly dressed twice a day, as the quantity, and indeed the quality, of the discharge from it required. The opening medicines were repeated occasionally, and his nights secured by a few drops of the Thebaic tincture.

In a few days, the sloughs from the edges of the *abdominal* muscles separated, and left the sore so largely open, that I could easily discover from whence the *fæces* made their *exit,* which was from the middle of that part of the *colon* that lies between the left kidney, to which it is attached, and the upper part of the *sacrum,* where it empties itself into, and forms the *rectum.*

It was exceedingly satisfactory and pleasing to observe, from day to day, the progress nature made in renovating this formidable breach, and her means of accomplishing it; for, after a little time, the surface of the intestines looked florid, and began to pullulate, throwing up small grains of flesh from every point. These *granules*, daily increasing, united with each other, and after filling up the intervals between the circumvolutions of the bowels, became one uniform surface; which surface meeting with that of the raw edges of the integuments, they both adhered together, and became one continued sore. As the wound incarned, the *fæcal* discharge lessened daily, and about the twenty-second or twenty-third day, entirely ceased. I now allowed him chicken-broth, milk-porridge, calves-feet-jelly, &c. The wound was dressed once a day with dry lint only, and in seven weeks it was completely healed.*

* In the 49th Volume of the Philosophical Transactions of London, page 238, we have "An account of a very remarkable case of a boy, who, notwithstanding that a considerable part of his intestines was forced out by the fall of a cart upon him, and afterwards cut off, recovered, and continues well." By John Nedham, of North-Walsham, Norfolk.

"The intestine cut off measured fifty-seven inches, by a string applied to the outer surface." The accident happened the 3d of January, 1755. On the 7th of May, the boy walked seven miles to dine with the surgeon—was perfectly well, and walked back again the same afternoon.

See also Mosely's account of the recovery of a young negro woman, after great protrusion of the intestines, from the Cæsarian operation performed by herself. And another case, more extraordinary, related by Mr. John Bell, of a soldier wounded by a halbert, who recovered, after walking a considerable distance with the protruded intestines wrapped up in his shirt, and placed in his hat.

